# Giant Clear Cell Renal Cell Carcinoma Masquerading as a Renal Cyst in a Dialysis Patient

**DOI:** 10.1016/j.xkme.2025.101137

**Published:** 2025-10-08

**Authors:** Yuki Oba, Ayaka Hane, Katsuyuki Miki, Kei Kono, Shinji Ito, Shigekazu Kurihara, Masayuki Yamanouchi, Tatsuya Suwabe, Yutaka Takazawa, Kenichi Ohashi, Yoshifumi Ubara, Yuki Nakamura, Naoki Sawa

**Affiliations:** 1 Nephrology Center, Toranomon Hospital Kajigaya, Kanagawa, Japan; 2 Department of Pathology, Toranomon Hospital, Tokyo, Japan; 3 Department of Human Pathology, Institute of Science Tokyo, Tokyo, Japan

An 85-year-old man treated with hemodialysis for 14 years had a renal cyst in his right kidney that gradually enlarged for 7 years. He underwent cyst punctures twice 4 years ago. At his third visit, cyst aspiration failed, so he was kept under observation. However, as the cyst enlarged and his abdominal swelling increased, he was referred to our hospital. The plain computed tomography showed that it was indeed a cyst, so he was admitted to our hospital for planned drainage inside the “cyst”.

**Figure 1:**
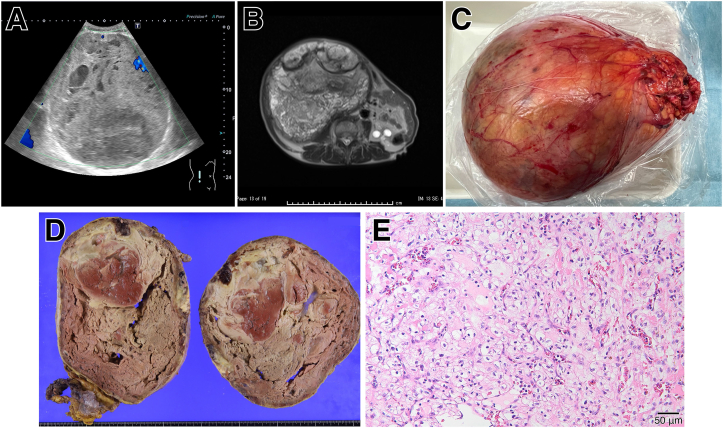
Radiologic and histologic findings of the right renal cell carcinoma. (A) Echographic image of a mass, (B) T2 weighted image of MRI, (C) The right kidney taken out by nephrectomy, (D) Bisection of the kidney showed filled with solid tumor, 22.0 × 21.0 × 16.0 cm, and (E) Hematoxylin-eosin staining of the carcinoma. Histopathology confirmed clear cell renal cell carcinoma emerged in an acquired cyst. Its pathological diagnosis was pT1bNx.

Echography before drainage showed solid components inside the cyst (Fig 1A), Thus, drainage was cancelled, and further imaging examination was performed. Contrast-enhanced computed tomography showed a mass on the cyst wall that was enhanced in the early phase. Magnetic resonance imaging showed unstructured solid components filled inside the mass (Fig 1B), suggesting cystic kidney cancer.

He underwent an elective right nephrectomy. The 4,400 g mass (Fig 1C) contained brown necrotic components and gray-white degeneration (Fig 1D). Histopathology confirmed clear cell renal cell carcinoma (RCC) emerged in an acquired cyst (Fig 1E). He was discharged without any postoperative complications and is still alive.

Renal cell carcinoma is often detected in an early stage, and the size of RCC has decreased thanks to the development and good access of imaging studies in the current era.^1^ However, sometimes giant RCCs are found, and they resemble cysts.^2^ Acquired renal cysts are common in patients treated with dialysis and have an increased risk of RCC.^3^ It is necessary to distinguish cystic renal masses from acquired renal cysts. It is important to perform a comprehensive imaging evaluation without being influenced by the patient’s medical history.
